# SGGly: a web server for whole-protein, structure-guided analysis of candidate N-linked glycosylation sites

**DOI:** 10.1093/nar/gkag507

**Published:** 2026-06-02

**Authors:** Xiaotong Gu, Yunzhuo Zhou, Yoochan Myung, David Ascher

**Affiliations:** School of Chemistry and Molecular Biosciences, University of Queensland, Brisbane, Queensland 4067, Australia; Australian Centre for Ecogenomics, The University of Queensland, Brisbane, Queensland 4067, Australia; Computational Biology and Clinical Informatics, Baker Heart and Diabetes Institute, Melbourne, Victoria 3004,Australia; School of Chemistry and Molecular Biosciences, University of Queensland, Brisbane, Queensland 4067, Australia; Australian Centre for Ecogenomics, The University of Queensland, Brisbane, Queensland 4067, Australia; Computational Biology and Clinical Informatics, Baker Heart and Diabetes Institute, Melbourne, Victoria 3004,Australia; School of Chemistry and Molecular Biosciences, University of Queensland, Brisbane, Queensland 4067, Australia; Australian Centre for Ecogenomics, The University of Queensland, Brisbane, Queensland 4067, Australia; School of Chemistry and Molecular Biosciences, University of Queensland, Brisbane, Queensland 4067, Australia; Australian Centre for Ecogenomics, The University of Queensland, Brisbane, Queensland 4067, Australia; Computational Biology and Clinical Informatics, Baker Heart and Diabetes Institute, Melbourne, Victoria 3004,Australia

## Abstract

N-linked glycosylation is critical for protein function and stability, yet identifying glycosylated sites remains challenging because glycosylation depends on sequence motifs and structural context. Many available computational approaches focus on motif-centred sequence windows and provide limited support for whole-protein inspection of candidate sites. SGGly is a freely accessible web server for structure-guided analysis of candidate N-linked glycosylation sites across full-length proteins. The server uses ProtBERT transformer-based embeddings with sequon and structure-derived residue descriptors to generate residue-level candidate-site predictions and returns downloadable residue-level predictions together with interactive 3D visualisation. Using a dual evaluation framework, SGGly achieved a Matthews correlation coefficient of 0.888 and receiver operating characteristic area under the curve of 0.987 under a strict, publication-supported regime. On the independent N-GlyDE benchmark, SGGly demonstrated strong generalisability, achieving the strongest specificity (0.941), sensitivity (0.993), and accuracy (0.946) among compared methods. SGGly provides a practical web resource for whole-protein glycosylation candidate mapping, structural inspection, and prioritisation of sites for follow-up analysis, guiding experimental design and interpreting glycoproteomic observations. SGGly is available at https://biosig.lab.uq.edu.au/sggly/. This website is free and open to all users, and there is no login requirement.

## Graphical Abstract

**Graphical Abstract:**
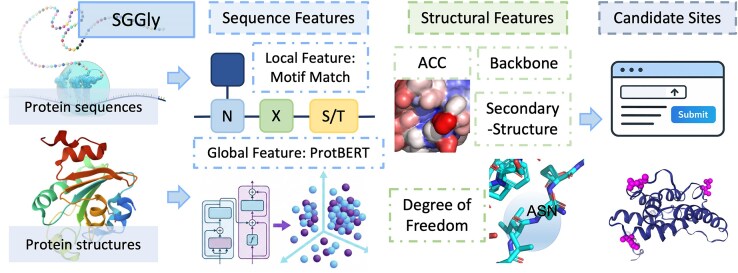

## Introduction

Protein glycosylation, a post-translational modification of proteins, occurs mainly in the lumen of the endoplasmic reticulum and plays a vital role in numerous biological processes, including protein folding, function, stability, and cellular signalling [1]. Among the various forms of glycosylation, N-linked glycosylation is one of the most prevalent in eukaryotes, and it involves the enzymatic addition of oligosaccharides (glycans) to the nitrogen atom of asparagine (Asn) residues within the consensus sequon Asn-X-Ser/Thr (where X can be any amino acid except proline) [2], but the presence of this motif alone is not sufficient to determine whether glycosylation occurs. Not all potential N-X-S/T sites are glycosylated, as structural accessibility, local conformation, and broader protein context also influence whether a sequon is likely to be modified. Given the significance of N-linked glycosylation in protein function, its alterations in numerous pathologies [1] and its association with various diseases like cancer [3], autoimmune disorders [4], Parkinson’s disease [5], and neurodegenerative conditions [6], practical tools for prioritising likely N-linked glycosylation sites remain valuable.

Experimental methods for identifying glycosylation sites, including mass spectrometry [7] and chromatography-based approaches [8], have laid the groundwork for identifying glycosylation sites but are often time-consuming and costly. Computational resources therefore play an important complementary role by helping users prioritise candidate sites for further study. Early predictors relied mainly on motif rules or sequence-derived features within fixed windows around candidate residues, and more recent methods have incorporated machine learning and deep learning to improve classification performance. For example, PTG-PLM [9] uses deep learning to predict glycosylation and glycation sites solely from sequence data, using protein language models trained on large-scale protein datasets. It applies transformer-based embeddings combined with convolutional neural networks to capture nuanced sequence patterns within specific windows around target motifs, achieving improved predictive accuracy by encoding the local sequence environment. However, many existing tools still focus on local motif-centred inputs, which can limit whole-protein interpretation and make it difficult to inspect candidate sites in their structural setting.

Recent advancements in protein structure prediction tools, like AlphaFold by DeepMind [10], have made it possible to describe candidate glycosylation sites using richer residue-level sequence and structural context. At the same time, high-quality predicted structures now support direct visual inspection of candidate residues in three-dimensional space. These developments are well-suited to web-based resources that allow users to analyse complete proteins rather than only pre-selected motif windows, and to inspect predicted candidate sites directly on the corresponding structure. SPRINT-Gly [11], for instance, integrated sequence-based and predicted structural features, such as half-sphere exposure, ASA, and secondary structural elements, using deep neural networks and support vector machines. SPRINT-Gly improved prediction of human and mouse glycosylation sites by integrating sequence-derived features within a machine-learning framework, with human N-linked sites receiver operating characteristic area under the curve (ROC-AUC) between 0.97 and 0.98. Pakhrin *et al*. [12] presented DeepNGlyPred, which uses sequence-based features of gapped-dipeptide and predicted structures to increase the specificity and sensitivity of glycosylation predictions by analysing evolutionary information and spatial constraints around potential glycosylation sites. They reported the Matthews correlation coefficient (MCC) and accuracy scores of 0.60 and 79.41%, respectively on the N-GlyDE [13] independent test set, recognising that structural accessibility and spatial context are useful determinants for glycosylation​​.

Here we describe SGGly, a web server for whole-protein, structure-guided analysis of candidate N-linked glycosylation sites. Whereas existing tools often focus on local sequence windows, SGGly enables residue-level whole-protein inspection in structural context through an accessible web interface. The server accepts protein structures through a PDB accession, a UniProt accession with AlphaFold structure retrieval, or uploaded PDB files, and reports residue-level candidate sites through downloadable tables and an interactive 3D viewer. SGGly integrates sequence context, sequon information, and structure-derived residue descriptors to support residue-level prioritisation across full-length proteins. We evaluated SGGly on low-redundancy UniProt-derived datasets and the independent N-GlyDE benchmark, which show that the server provides a practical resource for glycosylation candidate mapping and structural inspection at the whole-protein level.

## Materials and methods

### Datasets

The dataset used in this study was derived from reviewed human UniProt [14] entries with glycosylation annotations. UniProt CARBOHYD features were parsed to retain only valid N-linked glycosylation annotations on asparagine residues, while non-N-linked annotations, variant-specific annotations, and invalid sites were excluded. Each retained site was additionally labelled according to whether its UniProt evidence field contained PubMed-indexed experimental support or only annotation-based evidence without supporting publications. As AlphaFold [10] and DSSP [15] cannot reliably process sequences longer than 10 000 and 5000 residues, respectively, proteins exceeding 5000 residues were excluded. After preprocessing, the final dataset comprised 4414 proteins containing 15 633 retained N-linked glycosylation annotations, including 3041 PubMed-supported sites and 12 592 annotation-only sites. Thus, the majority of positive labels used in the broad UniProt-derived setting were annotation-only rather than directly supported by site-specific experimental publications. Accordingly, SGGly outputs should be interpreted primarily as candidate-site prioritisation, especially for proteins without prior experimental glycoproteomic characterisation. The final dataset contained 2 672 712 residues, 105 294 asparagine residues, and 21 246 canonical N-X-[not P]-S/T sequons. To address annotation-reliability concerns, we used a dual evaluation framework comprising a strict regime based on publication-supported positive sites and confident negatives, and a broad regime using all retained UniProt positive labels. In addition, separate analyses were performed on asparagine residues and on canonical sequon sites to provide evaluation views that were less imbalanced than residue-level assessment across all amino acids. Protein structures were retrieved from the AlphaFold Protein Structure Database using UniProt accessions. Where retrieved structures were inconsistent with UniProt residue numbering in labelled regions, replacement structure models were generated when required to improve sequence–structure consistency.

**Figure 1.**
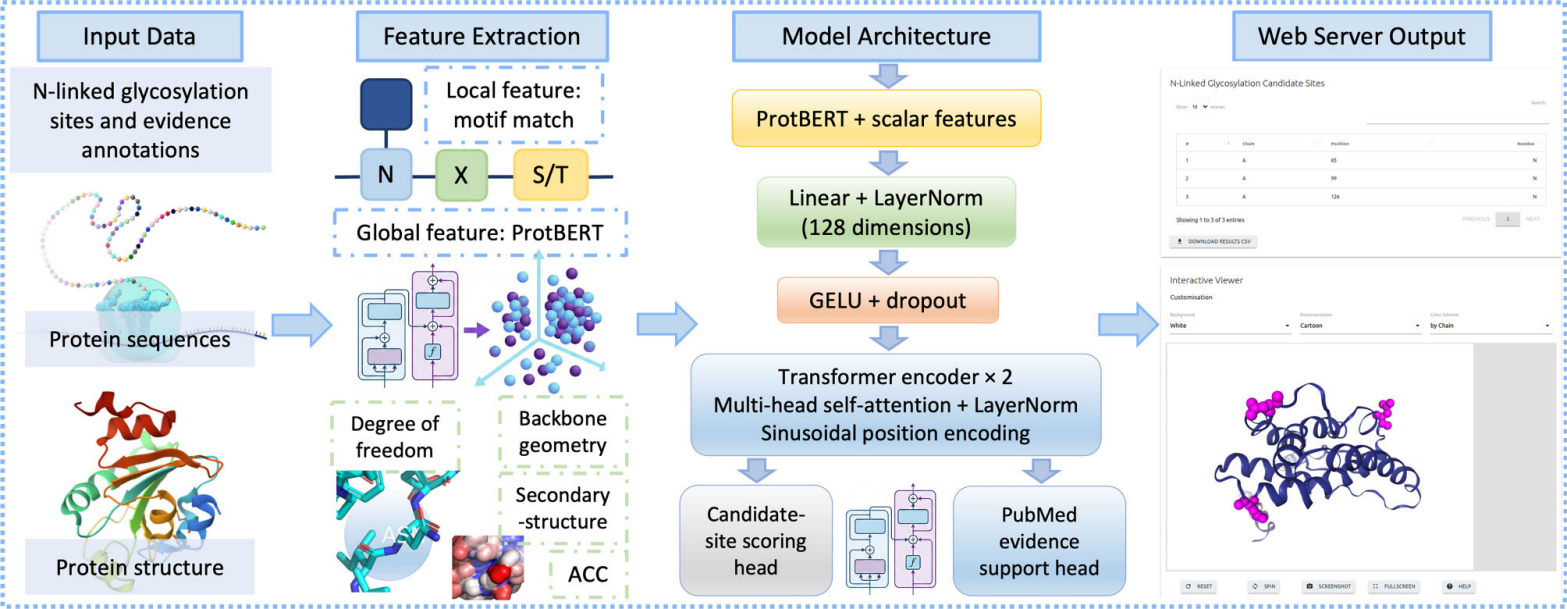
Overview of the SGGly server workflow. Protein sequences and structures are used to derive sequence- and structure-based residue-level descriptors, which are processed by the server to identify candidate N-linked glycosylation sites and return downloadable and visual outputs.

To reduce sequence redundancy and minimise information leakage, proteins were partitioned into low-redundancy training, validation, and test sets using whole-protein pairwise sequence similarity computed by global alignment, as described in the Supplementary Data. The final split comprised 3667 training proteins, 460 validation proteins and 287 test proteins, corresponding to 2 137 515, 267 710, and 267 487 residues, respectively. For independent external evaluation, we used the N-GlyDE dataset [13], which comprises 86 human proteins including both glycoproteins and non-glycoproteins. Full-length protein sequences were used in this study, and residue-level labels were assigned by UniProt accession and residue position. To avoid data leakage, proteins overlapping with the N-GlyDE benchmark were excluded from model training and validation before external testing. The overall SGGly workflow, including structure input, residue-level feature generation, candidate-site scoring and output visualisation, is summarised in Fig. 1.

### Server implementation

SGGly prioritises candidate N-linked glycosylation sites using a whole-protein transformer-based classifier applied to residue-level features derived from sequence context, sequon information, and protein structure. Residue-level sequence representations are generated using ProtBERT embeddings, while structure-based descriptors include DSSP-derived accessibility, backbone geometry, and secondary-structure features, together with AlphaFold confidence scores, spatial accessibility estimates, and structural match flags derived from structure models. This implementation enables whole-protein, residue-level scoring of candidate sites in structural context through the web interface, with results returned as downloadable tables and interactive structural visualisation. Additional details of feature construction, model architecture, training procedures, and score calculation are provided in the Supplementary Data.

#### Evaluation metrics

Predictive performance was assessed using accuracy, precision, recall, F1-score, specificity, balanced accuracy, MCC, Cohen’s kappa, ROC-AUC, and precision–recall area under the curve (PR-AUC). Because residue-level glycosylation prediction is imbalanced, MCC and PR-AUC were used as the most informative summary measures. Performance was evaluated across multiple residue views, including all residues, asparagine-only residues, and canonical sequon residues, with both all-label and PubMed-only evaluation settings. In addition, a dual evaluation framework was applied, comprising a strict regime using PubMed-supported positive sites against confident negatives and a broad regime using all retained UniProt positive labels against asparagine negatives. Additional threshold-dependent analyses are provided in the Supplementary Data.

### Web server

SGGly has been made accessible as a free web server hosted at https://biosig.lab.uq.edu.au/sggly/. The frontend is built using MaterializeCSS (version 1.0.0), while the backend functionality is powered by the Flask module (version 3.1.0) from Python 3.10. The web server is deployed through a WSGI application interface.

#### Input

SGGly is a web server for structure-guided prioritisation of candidate glycosylation sites. Single-protein jobs can be submitted by entering a valid PDB accession code, entering a UniProt accession code to retrieve the corresponding AlphaFold structure, or uploading a PDB file directly (Supplementary Manual S1). SGGly also supports batch submission of multiple PDB files in a single job through a dedicated Batch tab on the submission page; the prediction score threshold can be adjusted prior to submission, and results for all structures in the batch can be downloaded as a ZIP archive containing per-structure CSV files. Users may optionally provide an email address to receive notification upon job completion.

#### Output

Once the job is complete, the results page displays N-linked glycosylation candidate sites in a downloadable table together with an interactive 3D viewer for structural visualisation of the submitted protein and the identified residues. For experimental PDB structures containing repeated identical chains, results are reported for one representative chain per unique protein sequence unless a specific chain is selected by the user. The results table reports the following columns for each asparagine residue in the structure: Entry (job or structure identifier), Chain (chain identifier from the PDB file), Position (residue sequence number), Residue (amino acid identity), Score (continuous candidate score from 0 to 1 assigned by SGGly), and Candidate Status (‘Glycosylated’ for residues with a score of 0.5 or above, and ‘Not Glycosylated’ otherwise). The table supports searching, sorting and pagination, and can be exported as a CSV file for downstream analysis (Supplementary Manual S3). By default, residues with a prediction score of ≥0.5 are labelled as candidate sites on the server. Users seeking higher sensitivity may inspect lower-scoring residues, whereas users prioritising precision may apply a stricter threshold. The interactive viewer allows users to inspect predicted candidate residues directly on the protein structure; residues predicted above the threshold are highlighted in red. Display options include background colour, molecular representation, and colour scheme, as well as controls for spin, screenshot capture, and fullscreen mode (Supplementary Manual S2). Alongside the viewer, a Predicted Site Features panel reports structural context for each candidate residue: secondary structure assignment (helix, sheet or coil, derived from DSSP), solvent accessible surface area (ACC, in Å²), backbone dihedral angles phi and psi (in degrees), degree-of-freedom score (Freedom, a normalised measure of local structural permissiveness), and AlphaFold per-residue confidence (pLDDT) where available. Beyond displaying candidate residues, the interactive 3D viewer helps users assess whether a predicted site lies in a surface-accessible and structurally permissive environment, thereby supporting practical interpretation of the reported scores.

### Validation

We evaluated SGGly on the low-redundancy validation and test sets using full-length proteins and residue-level labels mapped by UniProt accession and residue position. Because N-linked glycosylation analysis is most relevant at candidate asparagine residues, the primary comparison focused on asparagine residues using all retained UniProt labels, as summarised in Table 1. Two whole-protein (WP) analysis branches were examined. Whole-Protein (All) uses the same full-protein sequence and structure context but is optimised across all residues, whereas Whole-Protein (Asn) retains the same whole-protein input context while restricting optimisation to asparagine residues. In this candidate-residue setting, both branches performed strongly, with Whole-Protein (Asn) giving the best overall balance of performance (MCC = 0.801, F1 = 0.818, precision = 0.699, recall = 0.985, ROC-AUC = 0.980, PR-AUC = 0.823), slightly outperforming Whole-Protein (All) (MCC = 0.793, F1 = 0.809, precision = 0.685, recall = 0.988, ROC-AUC = 0.979, PR-AUC = 0.820). These results indicate that focusing optimisation on candidate asparagine residues modestly improves prioritisation performance while preserving the same full-protein structural context.

**Table 1.** Performance of the two whole-protein SGGly branches on the low-redundancy test set

Analysis branch	Test-set setting	MCC	F1	Precision	Recall	ROC-AUC	PR-AUC
WP (All)	Asn residues, all retained labels	0.793	0.809	0.685	0.988	0.979	0.820
WP (Asn)	Asn residues, all retained labels	0.801	0.818	0.699	0.985	0.980	0.823
WP (All)	Strict regime	0.889	0.904	0.831	0.995	0.985	0.891
WP (Asn)	Strict regime	0.888	0.902	0.826	0.995	0.987	0.892
WP (All)	Broad regime	0.844	0.866	0.770	0.990	0.978	0.851
WP (Asn)	Broad regime	0.849	0.872	0.782	0.988	0.980	0.850

Primary results are shown for candidate asparagine residues using all retained UniProt labels; strict and broad regime results assess robustness to annotation uncertainty.

We additionally evaluated SGGly under two complementary label regimes (Table 1). In a strict regime, only PubMed-supported positive sites were treated as positives, while negatives were restricted to a conservative subset of structurally supported non-glycosylated asparagine residues. In a broad regime, all retained UniProt positive labels were used together with non-glycosylated asparagine residues. Under both regimes, performance remained strong for both whole-protein branches. The strict setting yielded the highest overall classification performance, whereas the broad setting was more challenging, consistent with the inclusion of annotation-only positives and a wider negative set. Together, these results support the interpretation of SGGly as a structure-guided system for prioritising candidate N-linked glycosylation sites for further inspection, rather than as a standalone binary site-calling tool.

### Independent N-GlyDE comparison

**Figure 2.**
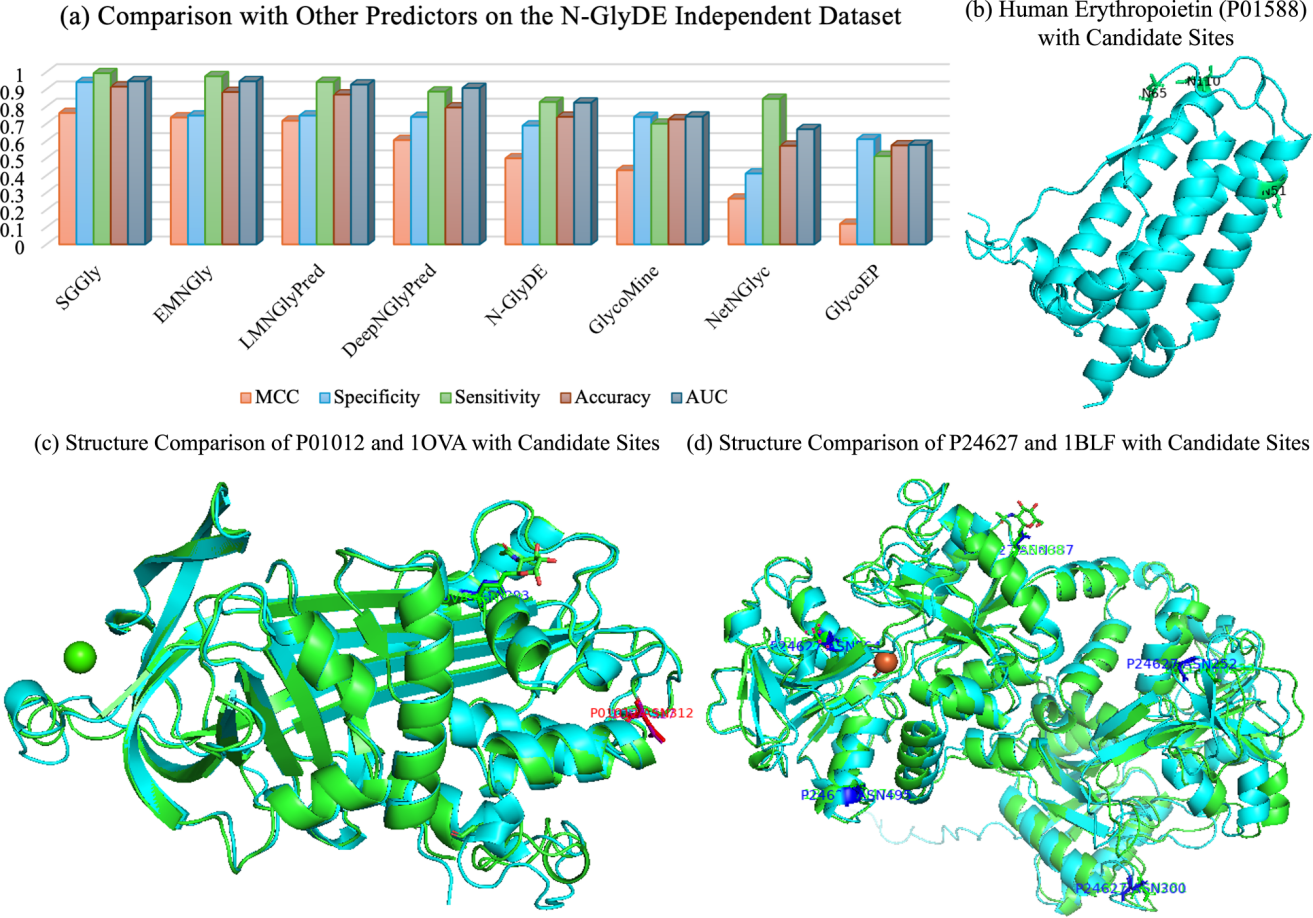
Comparison and structural examples for SGGly. (**a**) Comparison with other predictors on the N-GlyDE independent dataset. (**b**) Representative SGGly output. (**c**) AlphaFold–PDB comparison for P01012/1OVA. (**d**) AlphaFold–PDB comparison for P24627/1BLF.

For independent external benchmarking, SGGly was re-trained after excluding all proteins present in the N-GlyDE independent dataset from the training and validation sets, and was then evaluated on the benchmark using all asparagine residues, consistent with the candidate-residue setting used by the published comparison methods. Despite being trained only on glycosylated proteins and using whole-protein sequence and structure context rather than fixed local windows, SGGly showed strong generalisability on this independent dataset, achieving MCC = 0.762, specificity = 0.941, sensitivity = 0.993, accuracy = 0.946, and ROC-AUC = 0.979 at the default threshold. These results indicate that the whole-protein design remains effective even when evaluated on a benchmark that includes both glycoproteins and non-glycoproteins. Performance was even stronger when the analysis was restricted to benchmark glycoproteins only (MCC = 0.933, specificity = 0.978, sensitivity = 0.993, accuracy = 0.980, ROC-AUC = 0.984), supporting the view that the lower full-benchmark MCC partly reflects the inclusion of non-glycoproteins in the N-GlyDE dataset rather than a lack of discriminatory power on glycoprotein substrates. When only AlphaFold-derived models are available, predictions in high-confidence, well-ordered regions are more reliable, whereas predictions in low-pLDDT or flexible regions should be treated as prioritisation cues rather than definitive site calls. SGGly achieved strong sensitivity and overall benchmark performance while additionally providing whole-protein structural context and candidate-site visualisation through a public web server (Fig. 2a). Tool comparisons and references are discussed in detail in Supplementary Table S1.

### Biological application example

To illustrate the practical application of the SGGly web server, we analysed human erythropoietin (EPO), a well-characterised glycoprotein with multiple functionally important N-linked glycosylation sites. Submitting the AlphaFold structure of EPO to SGGly returned residue-level predictions across the full protein, identifying the experimentally validated glycosylation sites N51, N65, and N110 as high-confidence candidates (Fig. 2b). Other asparagine residues received substantially lower scores, consistent with less favourable local structural environments. Visualisation in the integrated three-dimensional viewer showed that the high-confidence sites were located on solvent-exposed surface regions, consistent with accessibility to the glycosylation machinery. In practice, the 3D viewer allows users to assess whether a positively scored sequon is surface-exposed, located in an ordered or disordered region, and spatially plausible for access by the glycosylation machinery. For example, a high-scoring residue that is buried within the protein core or positioned in a structurally constrained environment would be interpreted more cautiously than a similarly scored residue displayed on an exposed surface loop. The structural view therefore helps users distinguish scores that are supported by a plausible local environment from those that may warrant more careful follow-up. This example shows how the SGGly web server provides residue-level output in structural context, helping users distinguish likely glycosylated sequons from structurally constrained candidate sites and prioritise sites for experimental follow-up.

Although the training data were derived from reviewed human UniProt entries, the governing sequon rule and many structural determinants of N-linked glycosylation are not human-specific. Accordingly, we evaluated cross-species transfer using well-characterised non-human glycoproteins. We applied SGGly to chicken ovalbumin [16] (P01012/1OVA, Fig. 2c) and bovine lactoferrin [17] (P24627/1BLF, Fig. 2d), two well-characterised non-human glycoproteins. For ovalbumin, SGGly recovered the established glycosylation site corresponding to native Asn292 and also assigned a positive score to the second canonical sequon corresponding to the historically discussed Asn311 site. Because this site is generally not occupied in native egg-white ovalbumin [18], we interpret this result as highlighting latent glycosylation permissiveness at a plausible sequon rather than definitive occupancy in the native state. By contrast, bovine lactoferrin showed strong concordance with experimental knowledge. SGGly recovered all five widely reported bovine lactoferrin N-glycosylation sites, and predictions from the full-length P24627 structure and the experimental 1BLF structure were concordant after accounting for mature-protein numbering. Notably, the site corresponding to Asn281 received the lowest positive score among the five sites, which is biologically consistent with reports that this site is less heavily occupied than the other four bovine lactoferrin glycosylation sites [17].

## Conclusion

SGGly provides a freely accessible web server for whole-protein, structure-guided analysis of candidate N-linked glycosylation sites. In contrast to approaches limited to motif-centred sequence windows, SGGly enables analysis across full-length proteins and returns residue-level downloadable outputs together with integrated three-dimensional structural visualisation. By combining a whole-protein transformer-based classifier with structure-derived descriptors, tabulated results, and structural inspection within a single workflow, the server supports practical exploration and prioritisation of candidate sites without requiring users to combine multiple separate tools.

Evaluation on low-redundancy UniProt-derived test sets showed that SGGly performs strongly on publication-supported glycoproteins, achieving an ROC-AUC of 0.987 and an F1-score of 0.902 in the strict asparagine-residue setting, while maintaining good performance on annotation-only proteins. On the independent N-GlyDE benchmark, SGGly achieved competitive performance relative to existing approaches, with the strongest specificity (0.941), sensitivity (0.993), and accuracy (0.946) among the compared methods. These results support the practical utility of the server for residue-level glycosylation candidate mapping across full-length proteins.

SGGly is intended to complement experimental glycoproteomics by helping users identify, inspect, and prioritise candidate N-linked glycosylation sites for further study. Importantly, in the UniProt-derived data used here, the majority of retained positive annotations are annotation-only rather than supported by site-specific experimental publications. The server is particularly useful for proteins in which experimentally annotated glycosylation sites remain limited. As with other structure-guided approaches, performance depends on the quality of the underlying protein structure models. When only AlphaFold-derived models are available, users should place greater confidence in predictions from high-pLDDT, well-ordered regions and treat sites in low-confidence or flexible regions as prioritisation cues rather than definitive calls. Future updates may expand dataset coverage and further improve structural handling for challenging proteins. SGGly is freely available at https://biosig.lab.uq.edu.au/sggly/.

## Supplementary Material

gkag507_Supplemental_File

## Data Availability

This website is free and open to all users, and there is no login requirement. The SGGly web server is available at https://biosig.lab.uq.edu.au/sggly/.
