# Supplementary material for: SGGly: a web server for whole-protein, structure-guided analysis of candidate N-linked glycosylation sites
Source: Nucleic Acids Res. 2026 Jun 2;54(W1):W314–20. doi: 10.1093/nar/gkag507 (PMC13355084; doi:10.1093/nar/gkag507)
Supplement: gkag507_Supplemental_File [file gkag507_supplemental_file.docx]

**SUPPLEMENTARY MATERIAL**

# **SGGly: a web server for whole-protein, structure-guided analysis of candidate N-linked glycosylation sites**

Xiaotong Gu^1,2,3^, Yunzhuo Zhou^1,2,3^, Yoochan Myung^1,2^, David Ascher^1,2,3,*^

^1^ School of Chemistry and Molecular Biosciences, University of Queensland, Brisbane, Queensland, Australia

^2^ Australian Centre for Ecogenomics, The University of Queensland, Brisbane, Queensland, Australia

^3^ Computational Biology and Clinical Informatics, Baker Heart and Diabetes Institute, Melbourne, Victoria, Australia

^*^ To whom correspondence should be addressed. Tel: +61 90354794; Email: d.ascher@uq.edu.au

**Representative N-linked glycosylation prediction tools**

Table S1 summarises representative N-linked glycosylation site prediction tools published between 2019 and 2025, comparing their input scope, main representation, use of structural information, public availability and server-side visualisation. Most earlier methods are based on fixed sequence windows around candidate sites, whereas more recent approaches increasingly incorporate full-length protein language model embeddings and, in some cases, structure-derived features. Here, “structure used” refers to either explicit structural descriptors or features derived from pretrained structure models rather than interactive structural inspection. Public availability was checked against publicly accessible sources in April 2026.

**Low-redundancy dataset partitioning**

To construct low-redundancy data partitions, whole-protein pairwise sequence similarities were calculated using global sequence alignment implemented using the PairwiseAligner module from Biopython (1). Alignment scores were computed using a permissive scoring scheme (match = 1, mismatch = 0, gap open = 0, gap extend = 0) and normalised by the length of the longer sequence to obtain a similarity value for each protein pair. For each sequence, the minimum, median and maximum similarity values across the dataset were summarised for each protein. Proteins were ranked from lowest to highest similarity and assigned greedily to the test set, then the validation set, and finally the training set until approximate residue-budget targets of 10%, 10% and 80% were reached. This produced final partitions of 3,667 proteins for training, 460 for validation and 287 for testing, corresponding to 2,137,515, 267,710 and 267,487 residues, respectively. The training, validation and test sets contained 12,515, 1,677 and 1,441 positive N-linked sites, including 2,365, 381 and 295 PubMed-supported sites, respectively. Median maximum similarity to the training set was 0.3873 for validation proteins and 0.3767 for test proteins, while median train-reference median similarity was 0.2708 and 0.2416, respectively. Figure S1 shows the distributions of minimum, median and maximum similarity scores for validation and test proteins relative to the training set.


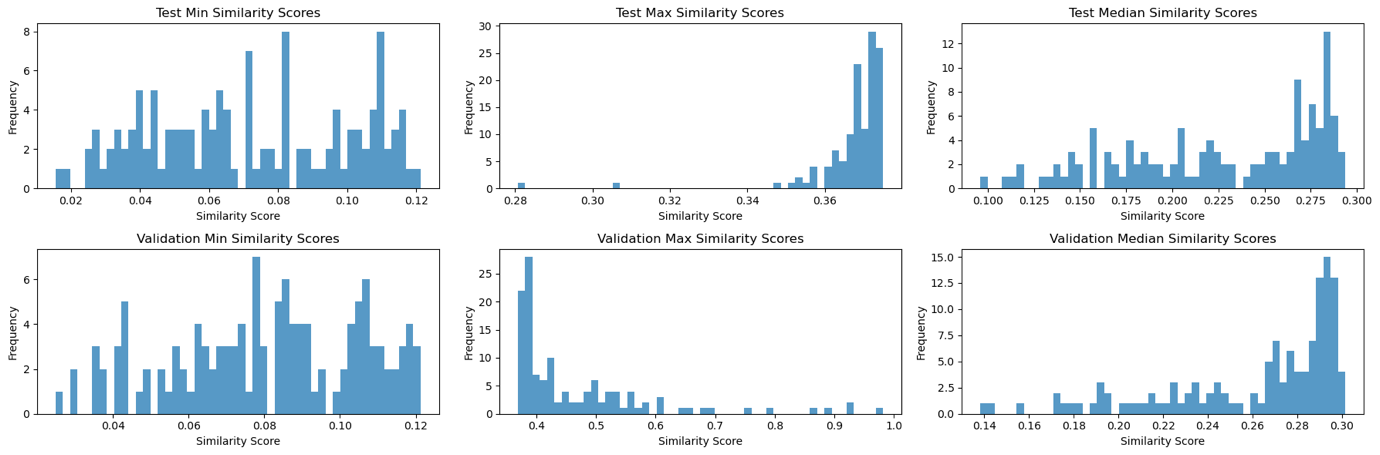


Figure S1. Distribution of minimum, median, and maximum whole-protein similarity scores for test (top) and validation (bottom) proteins relative to the training set, calculated using global sequence alignment.

**N-linked glycosylation site prediction tools comparison**

Supplementary Table S1 summarises representative N-linked glycosylation site prediction tools reported between 2019 and 2025. Most methods are based on fixed sequence windows centred on candidate residues, and many do not use explicit residue-level structural context from full-protein models. Several tools are available only as code repositories or were reported without a currently accessible public server, and standard outputs are typically limited to tabular predictions without structural visualisation of candidate sites. In contrast, SGGly performs whole-protein residue-level scoring using integrated sequence and structure descriptors, is accessible through a public web server, and presents predicted candidate sites directly in their structural context for interactive inspection. This distinction is important because the server is intended not only to rank candidate residues, but also to support biological interpretation of those candidates within the full protein structure.

**Feature generation and definition of the degree-of-freedom score**

Table S1. Representative N-linked glycosylation site prediction tools (2019–2025)

| **Tool** | **Year** | **Input scope** | **Main representation** | **Structure used** | **Public availability** | **Server visualisation** |
| --- | --- | --- | --- | --- | --- | --- |
| SPRINT-Gly (2) | 2019 | Sequence window | Handcrafted sequence/evolutionary/predicted-structure features + DNN/SVM | Yes - predicted structural properties | Web server and GitLab code | No - standard text/tabular output |
| N-GlyDE (3) | 2019 | Sequence window | Two-stage model: protein-similarity voting + pattern/gapped-dipeptide SVM | No | Web server reported in paper; no public code repository identified | No - standard web prediction output |
| N-GlycoGo (4) | 2020 | Sequence window | Handcrafted multi-encoding features + XGBoost | Yes - engineered structure-related descriptors, not coordinate-based | Web server reported in paper but not available; no public code repository identified | N/A |
| Nglyc (5) | 2020 | Sequence window | Handcrafted sequence features + random forest | No clear explicit structure use identified from public sources | GitHub code only; no public web server identified | N/A |
| DeepNGlyPred (6) | 2021 | Sequence window | Handcrafted sequence/evolutionary/predicted-structure features + MLP | Yes - predicted structural properties | GitHub code only | N/A |
| PUStackNGly (7) | 2022 | Sequence window | Handcrafted multi-encoding features + PU-stacking ensemble | Yes - engineered structure-related descriptors | GitHub code only | N/A |
| LMNglyPred (8) | 2023 | Full-length sequence embeddings, site-level scoring (sequon-restricted) | PLM embeddings + deep classifier | No | GitHub code only | N/A |
| EMNGly (9) | 2023 | Full-length sequence/structure embeddings, site-level scoring | PLM + structure-model embeddings + SVM | Yes - AF2-predicted 3D coordinates fed into ESM-IF1 | GitHub code only; no public web server identified | N/A |
| N-GlycoPred (10) | 2024 | Sequence window | One-hot/AAindex + CNN-attention-BiLSTM | No | No public server or code repository identified | N/A |
| CoNglyPred (11) | 2025 | Full-length sequence + structure, site-level scoring | ESM-2 embeddings + structural graph + co-attention | Yes | GitHub code only | N/A |
| StackGlyEmbed (12) | 2025 | Full-length sequence embeddings, site-level scoring | Multi-PLM embeddings + stacking ensemble | No | GitHub code only | N/A |
| ESM-LoRA-Gly (13) | 2025 | Full-length sequence embeddings, site-level scoring | Fine-tuned ESM2-3B (LoRA) | No | Preprint only; GitHub code only; no public web server | N/A |

Sequence- and structure-based features were generated for each residue, producing 12 scalar features plus 1024-dimensional ProtBERT embeddings per residue. Structural features were obtained from DSSP files generated using mkdssp (14) from AlphaFold-predicted protein structure models. From these files, residue-level annotations including solvent accessibility (ACC), backbone torsion angles (PHI, PSI), and secondary structure type were extracted. Secondary structure was encoded as three binary features (SS_H, SS_E, SS_C) using one-hot encoding of DSSP assignments: H, G, and I were mapped to helix (H); E and B were mapped to strand (E); all others were mapped to coil (C). The AlphaFold pLDDT confidence score was extracted from the B-factor field of CA atoms. Two binary flags of DSSP match and pLDDT match indicated whether structural data was available for each residue. Sequence-based features included a binary indicator for the canonical N-linked glycosylation sequon, N-X-[not P]-S/T, a sequon context score encoding the identity of the +2 position (threonine = 1.0, serine = 0.5, other = 0.0), and ProtBERT residue embeddings extracted from the full-length protein sequence. C$\alpha$ coordinates were used where required for spatial feature calculation.

In addition to DSSP-derived features, a degree-of-freedom score was computed for every residue in the protein to characterise local structural permissiveness. Let $N$ denote the set of asparagine residues and $R$ is the set of all residues in the protein. For each asparagine residue $i\in N$, located at coordinates $\left( x_{i} , y_{i} , z_{i} \right)$, neighbouring residues within a radius of 10 Å were identified using C$\alpha$ coordinates. The degree-of-freedom score was defined as the inverse of the sum of inverse Euclidean distances to neighbouring residues within this threshold:

$$F_{i}=\left\{ \begin{matrix} \left( \sum_{\begin{aligned} j\in R, j\neq i \\ d_{ij\leq10Å} \end{aligned}} \frac{1}{d_{\mathrm{ij}}} \right)^{-1}, & \text{if at least one neighbouring residue is present} \\ 1, & \text{otherwise} \end{matrix} \right.$$

where the Euclidean distance between residues $i$ and $j$ is

$$d_{ij}=\sqrt{\left( x_{i}-x_{j})^{2}+(y_{i}-y_{j})^{2}+(z_{i}-z_{j})^{2} \right.}$$

The raw degree-of-freedom scores were normalised as

$$F_{i}^{\mathrm{norm}}=\frac{F_{i}}{F_{\max}}$$

where $F_{\max}$is the maximum freedom score observed across the dataset. Non-asparagine residues were assigned a score of 0, as they were not considered candidate N-linked glycosylation sites.

Model training details

SGGly was implemented as a whole-protein transformer-based classifier trained on residue-level sequence and structure features. The input representation for each residue comprised ProtBERT embeddings (1024 dimensions) together with 12 scalar features capturing sequon information and structure-derived descriptors. These scalar features included the canonical sequon indicator and context, DSSP-derived accessibility and backbone geometry, secondary-structure one-hot encoding, freedom score, pLDDT, and binary flags indicating whether DSSP and pLDDT information were available. The combined feature vector was projected to a 128-dimensional latent space using a linear layer followed by layer normalisation, GELU activation and dropout (0.2), after which the residue representations were processed by a two-layer TransformerEncoder with four attention heads and sinusoidal positional encoding. The network used two prediction heads, one for glycosylation classification and one for PubMed-support evidence, reflecting the multi-task training setup used in the revised pipeline.

Training was performed using a memory-efficient pipeline designed for whole-protein inputs. Low-redundancy train, validation and test sets were constructed by ranking proteins using whole-protein pairwise sequence similarity computed by global alignment, and proteins longer than the maximum training length were split into non-overlapping windows during optimisation, while validation and test proteins were retained at full length. To reduce memory usage, the pipeline used mmap-backed loading of the feature arrays, length-binned batching, automatic mixed precision, and gradient checkpointing. The optimisation scheme used AdamW with a learning rate of 3 × 10⁻4 and weight decay of 1 × 10⁻4, together with gradient clipping at 1.0 and a ReduceLROnPlateau scheduler. A weighted binary cross-entropy objective was used for glycosylation prediction, and the PubMed-support head was included with a loss weight of 0.2. The positive-class weight was estimated from the training set and capped to maintain stable optimisation. Training proceeded for up to 150 epochs, with early stopping based on validation performance, and the checkpoint with the best validation criterion was retained as the final model. The trained model contains approximately 5.46 × 10⁵ parameters.

Threshold performance, precision–recall behaviour and training dynamics

Figures S2a–c show the threshold analysis, precision–recall curves and training dynamics, respectively, and together indicate that SGGly is stable and performs consistently across a range of operating conditions. In Figure S2a, the threshold analysis shows that the main classification metrics change gradually across thresholds, suggesting that the model is robust rather than overly sensitive to a single cut-off. In particular, recall remains high while precision increases more noticeably at stricter thresholds, making the trade-off between sensitivity and selectivity clear for users. In Figure S2b, the precision–recall curves show strong discrimination on the test set, with PR-AUC values of 0.820 for the whole-protein model and 0.823 for the Asn-focused model, indicating that both branches maintain useful performance under class imbalance. Finally, Figure S2c shows that both models converge stably during training, with early stopping selecting sensible epochs and validation MCC and ROC AUC remaining consistently high, which supports the reliability of the learned representations and the overall training procedure.


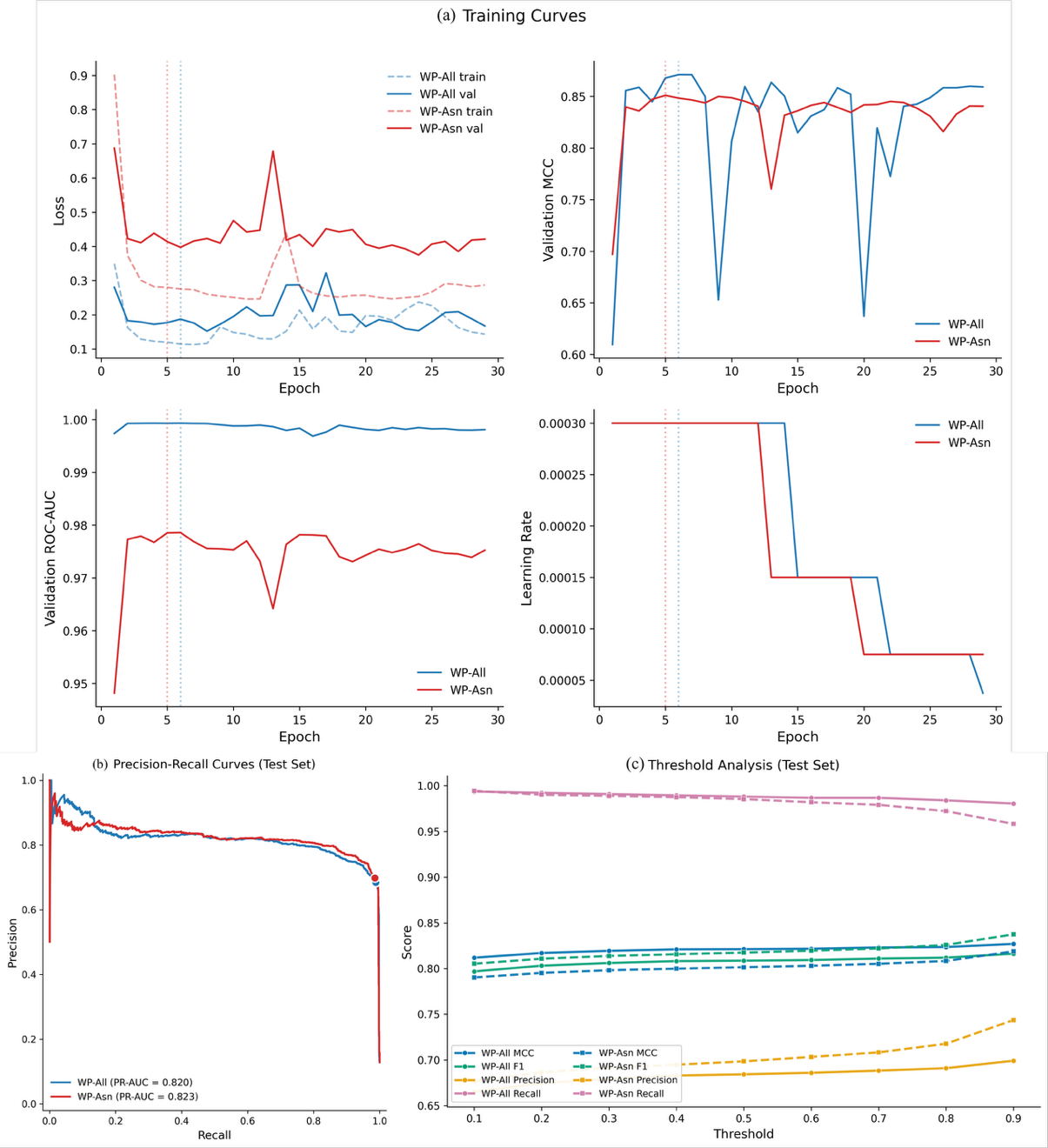


Figure S2. Threshold analysis, precision–recall curves and training dynamics for the whole-protein and Asn-focused SGGly models. (a) Test-set performance across decision thresholds. (b) Precision–recall curves on the test set. (c) Training curves with early stopping.

Ablation analysis

A single-feature ablation analysis showed that the full SGGly model achieved the strongest overall performance, with the sequon group alone retaining much of the predictive signal, consistent with the established importance of the canonical N-X-[not P]-S/T motif in N-linked glycosylation. ProtBERT alone captured useful full-protein sequence context but remained inferior to the full integrated model, indicating that whole-protein embeddings are informative yet benefit from explicit motif and structural descriptors. The individual structural feature groups had smaller standalone effects, but they still contribute complementary information on accessibility, local geometry, confidence and structural completeness. This pattern is consistent with the biology of N-linked glycosylation, which occurs mainly during cotranslational entry into the endoplasmic reticulum, while still allowing post-translocational modification at some sites, so structural descriptors are likely most useful as contextual refinements rather than dominant predictors in isolation.

External benchmark dataset

The N-GlyDE dataset (3) was originally constructed as an experimentally curated benchmark for N-linked glycosylation prediction. In the original study, annotations were assigned only to N-X-S/T sequons supported by experimental evidence, while sequons labelled as “Probable”, “Potential” or “By sequence similarity” were excluded. This filtering strategy was consistent with the original sequence window-based design of N-GlyDE, in which only local regions surrounding candidate motifs were used for model input.

In contrast, the present study used full-length protein sequences and assigned residue-level labels through UniProt annotations linked by UniProt accession. Because SGGly was trained on a separate UniProt-derived dataset rather than the original N-GlyDE training set, proteins associated with the N-GlyDE benchmark were removed from model development wherever overlap was identified by UniProt accession and protein sequence.

Supplementary note on the N-GlyDE non-glycoprotein subset

The N-GlyDE independent dataset includes 33 non-glycoproteins selected from subcellular locations such as the nucleus, cytosol and mitochondrion, where N-linked glycosylation is not generally expected to occur. Review of UniProt subcellular localisation annotations showed that 17 of these proteins lacked annotation to glycosylation-associated compartments, such as the endoplasmic reticulum, Golgi apparatus, secreted or membrane regions, whereas the remaining 16 proteins had at least one such annotation. Because subcellular localisation was beyond the scope of model development, these 17 proteins were excluded in an additional sensitivity analysis. After exclusion, SGGly achieved an MCC of 0.811 on the remaining independent subset.

**Manual S1. Running analysis for single protein N-linked glycosylation candidate sites**

**
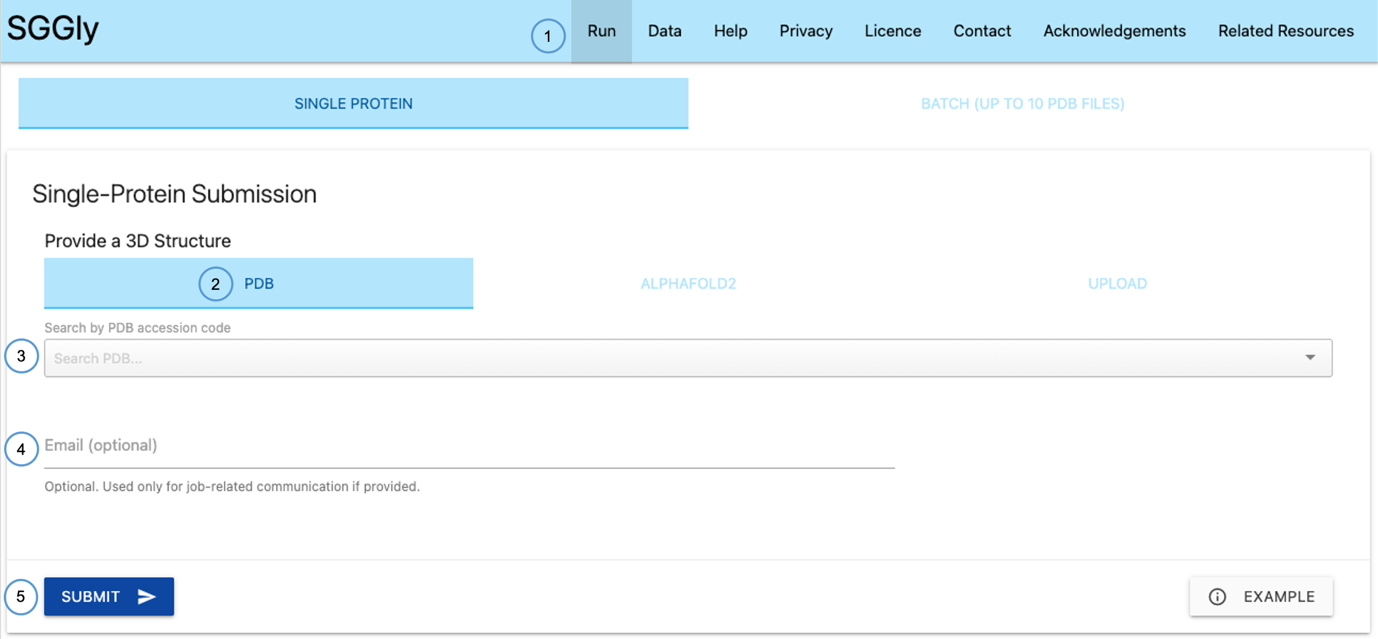

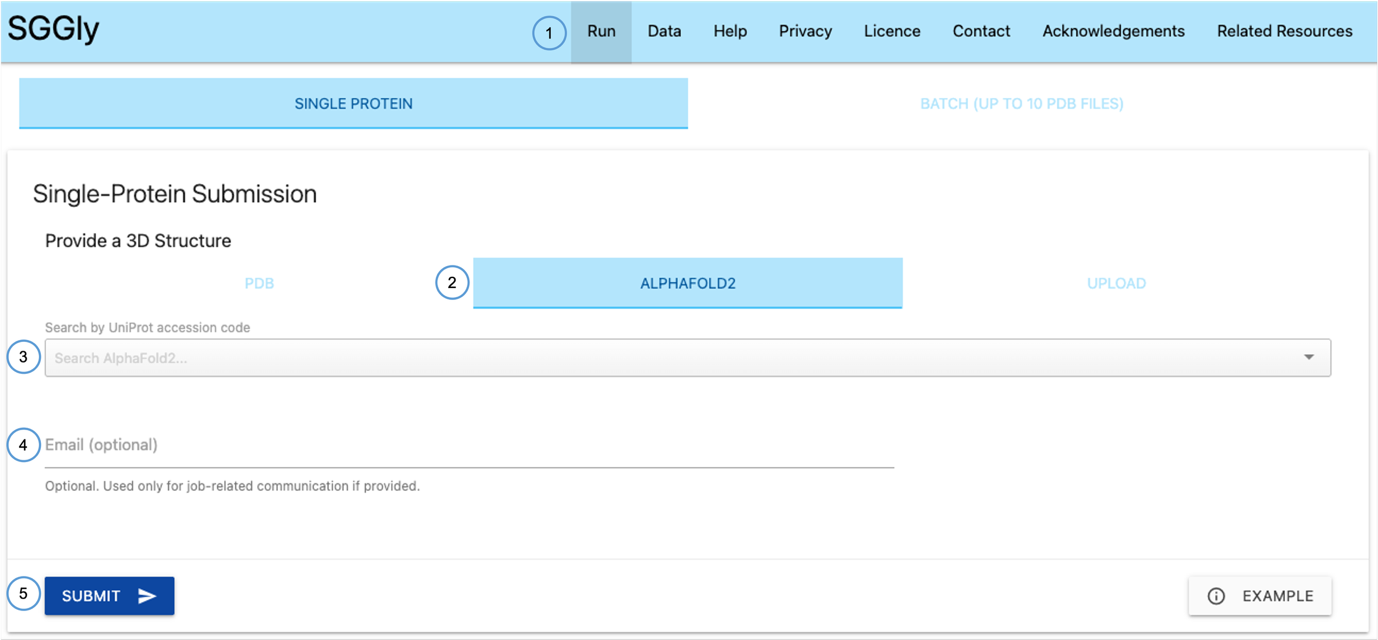

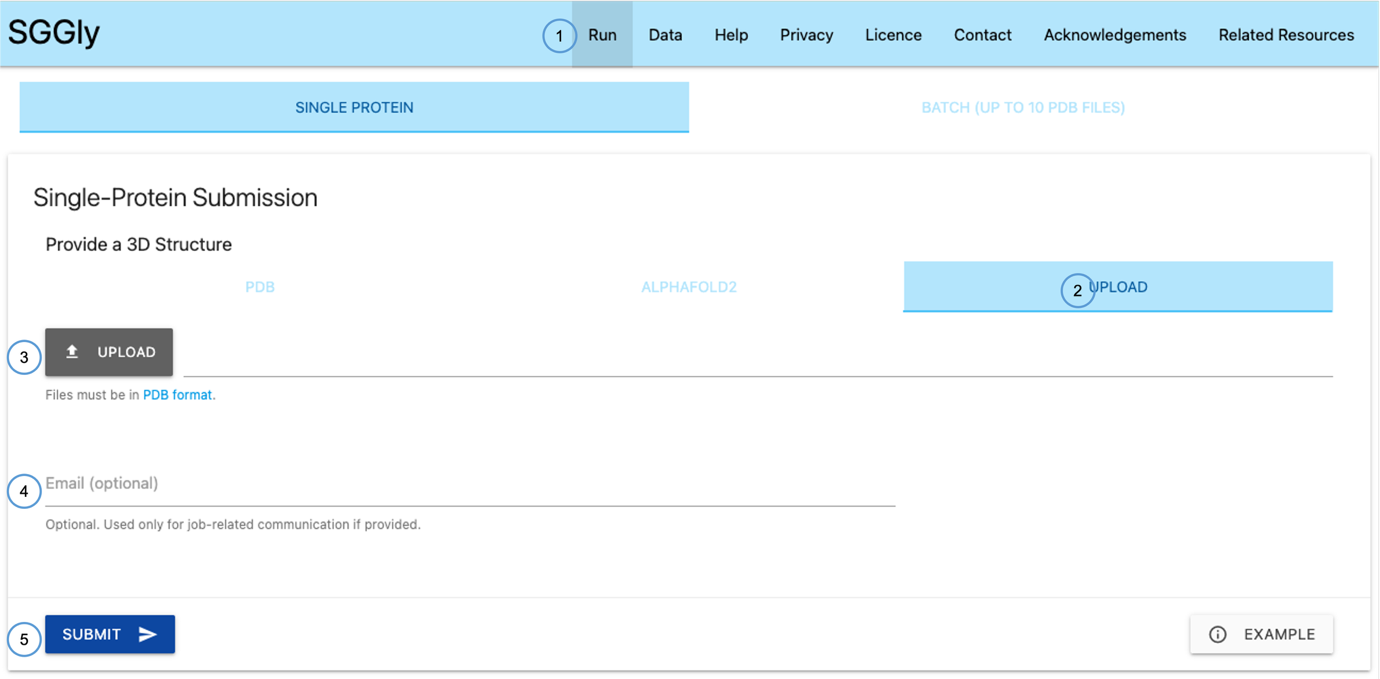
**

**
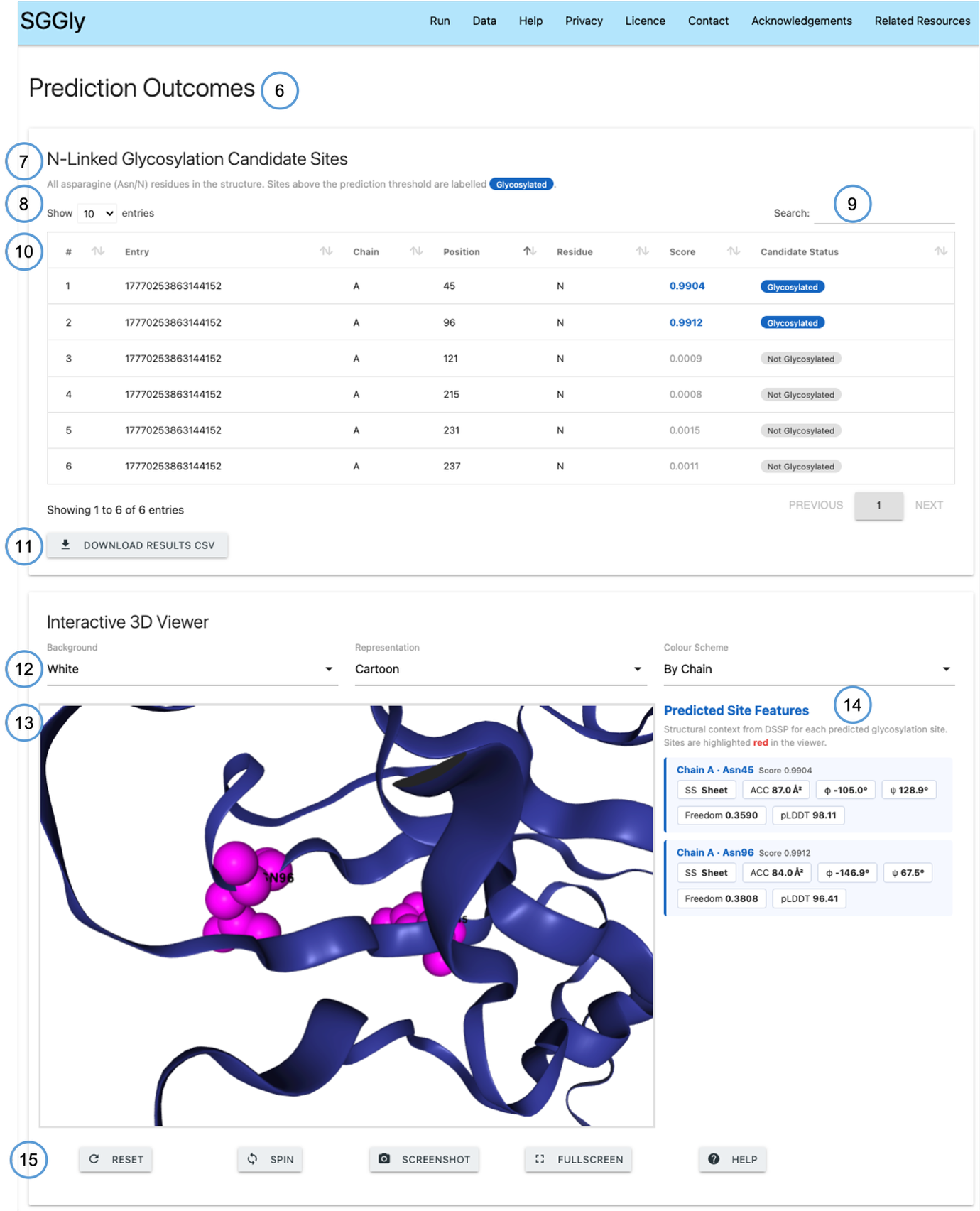
**

The submission page can be accessed via the menu item Run (1) on the top bar menu.

SGGly provides three options for supplying a protein structure:

- The PDB option (2) allows the user to provide a structure by entering a valid PDB accession code.
- The AlphaFold2 option (2) allows the user to provide a structure by entering a UniProt accession code for retrieval of the corresponding AlphaFold model.
- The Upload option (2) allows the user to upload a structure file in PDB format.

If provided, an email address (3) can be used to notify the user when the job has finished.

The Submit button (4) is used to run the analysis.

An Example button (5) is also available at the bottom of the form to display a sample results page.

**Manual S2. Running analysis of multiple proteins with batch**

**
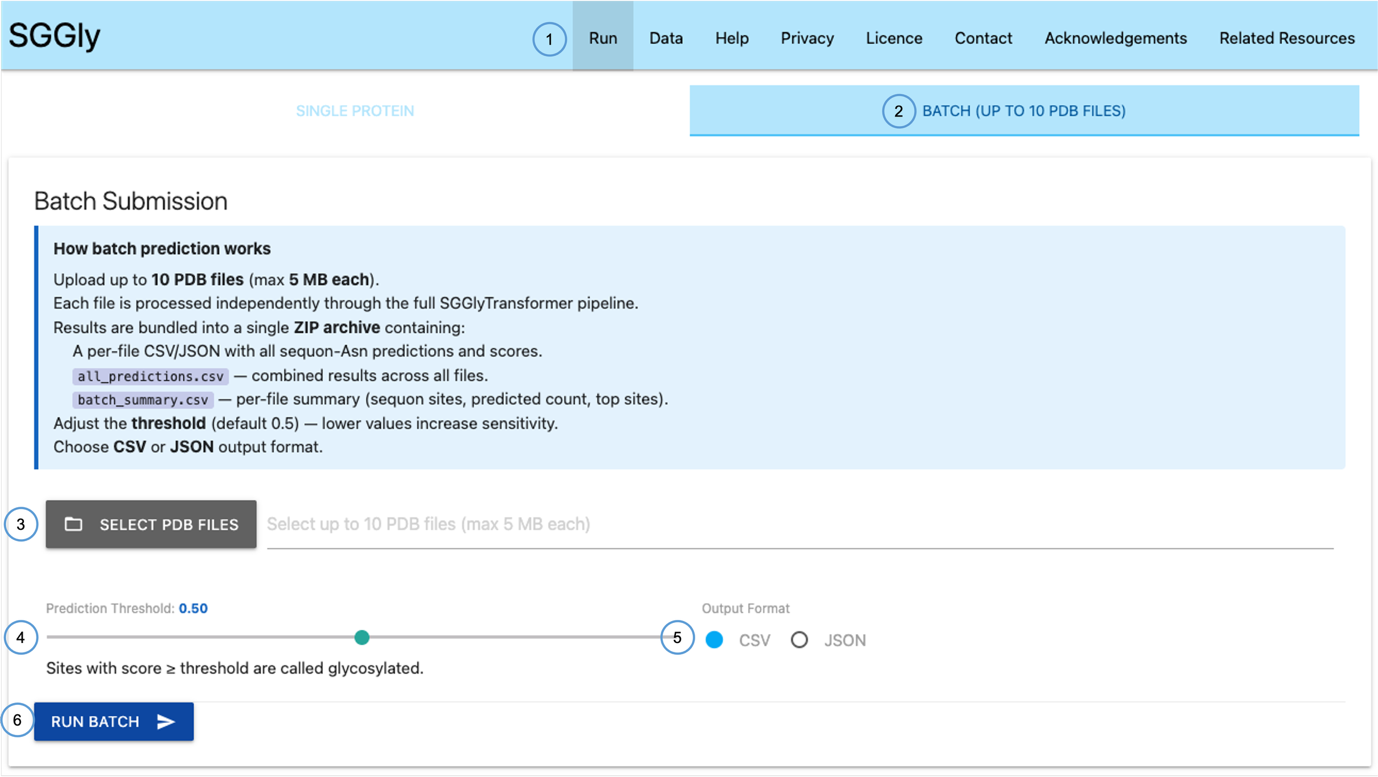
**

**
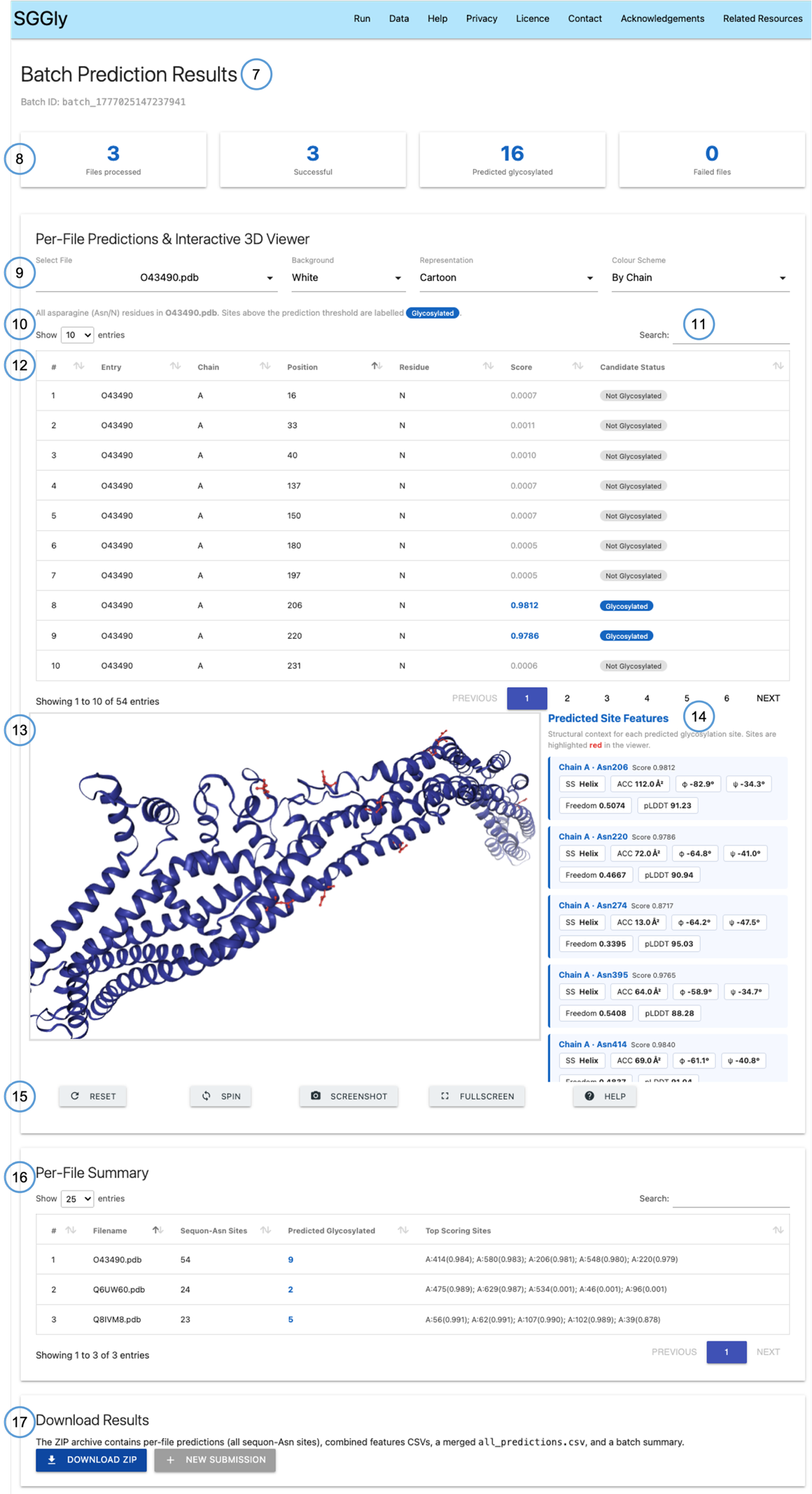
**

The Batch tab on the submission page allows users to submit multiple structure files in a single job. Files must be in PDB format and are selected using the file upload control (3). The prediction score threshold used to classify candidate sites can be adjusted using the threshold slider (4), and the output file format for the downloadable archive can be selected as CSV or JSON (5). Clicking Run Batch (6) submits all selected files for processing. Once the batch job is complete, the results page displays an overview of all submitted structures together with a per-file interactive viewer and summary table. A Download ZIP button allows the user to export prediction result files for all structures in the batch.

For each protein in a batch submission, once prediction is complete, the corresponding results view displays two main panels.

- The Prediction Outcomes panel (6) displays a table (7) of N-linked glycosylation candidate sites for the submitted structure. The table reports the following columns: Entry (the submitted job or structure identifier), Chain (chain identifier from the PDB file), Position (residue number in the structure), Residue (amino acid identity), Score (the candidate score assigned by SGGly, ranging from 0 to 1), and Candidate Status (“Glycosylated” for residues with a score of 0.5 or above, and “Not Glycosylated” otherwise). Standard controls for pagination, column sorting and text search are provided.
- A Download Results CSV button (8) allows the user to export the results as a comma-separated values file.
- The Interactive Viewer panel (9) displays the input protein structure with predicted candidate residues highlighted.
- A set of viewer customisation controls (10) is provided, including options such as background, representation and colour scheme.
- Action buttons at the bottom of the viewer (11) allow the user to reset the view, spin the structure, take a screenshot, switch to fullscreen mode, or open the help panel.
- A Predicted Site Features panel alongside the viewer displays structural context for each residue predicted above the threshold. For each candidate site the panel reports: secondary structure assignment (Helix, Sheet or Coil, derived from DSSP); solvent accessible surface area (ACC, in Å²); backbone dihedral angles (φ and ψ, in degrees); degree-of-freedom score (Freedom), a normalised measure of local structural permissiveness; and AlphaFold per-residue confidence (pLDDT), where available. Candidate sites are highlighted in the 3D viewer.

**Manual S3. Downloaded results file example**

**
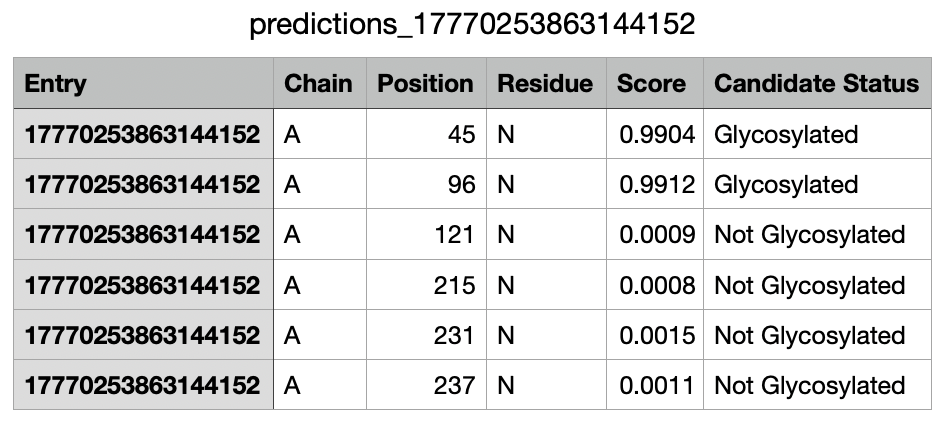
**

Results can be downloaded directly from the results page by clicking the Download Results CSV button (8). The downloaded file is a comma-separated values (CSV) file containing residue-level predictions for all asparagine residues in the submitted structure. An example filename takes the form predictions_<job_id>.csv, where <job_id> is the unique identifier assigned to the submitted job. The file contains the following fields:

- Entry: the unique job identifier assigned to the submission, or the structure identifier for batch jobs
- Chain: chain identifier from the submitted PDB file
- Position: residue sequence number as recorded in the PDB file
- Residue: amino acid identity at this position (asparagine for all reported candidates)
- Score: continuous candidate score assigned by SGGly, ranging from 0 to 1; residues with a score of 0.5 or above are classified as candidate glycosylation sites by default
- Candidate Status: classification label assigned by SGGly; “Glycosylated” indicates a score at or above the threshold (default 0.5), and “Not Glycosylated” indicates a score below the threshold

In addition to the downloadable CSV, the results page displays a Predicted Site Features panel for each residue predicted above the threshold. This panel reports the following structural descriptors derived from the submitted structure: secondary structure assignment (Helix, Sheet or Coil, computed using DSSP); solvent accessible surface area (ACC, in Å²); backbone dihedral angles phi (φ) and psi (ψ) in degrees; degree-of-freedom score (Freedom), a normalised measure of local structural permissiveness computed from inter-residue distances; and AlphaFold per-residue confidence (pLDDT), reported where available. These descriptors are displayed on the results page and are intended to support manual inspection of each candidate site in its structural context.

**Batch download**

For batch submissions, a Download ZIP button on the batch results page allows the user to export prediction result files for all structures processed in the job. The ZIP archive contains one CSV file per submitted structure. Each file follows the same column format described above (Entry, Chain, Position, Residue, Score, Candidate Status) and is named predictions_<structure_name>.csv.

Reference

1. Cock, P.J., Antao, T., Chang, J.T., Chapman, B.A., Cox, C.J., Dalke, A., Friedberg, I., Hamelryck, T., Kauff, F., Wilczynski, B. *et al.* (2009) Biopython: freely available Python tools for computational molecular biology and bioinformatics. *Bioinformatics (Oxford, England)*, **25**, 1422–1423.

<http://www.ncbi.nlm.nih.gov/pubmed/19304878>

<http://dx.doi.org/10.1093/bioinformatics/btp163>

<http://www.ncbi.nlm.nih.gov/pmc/articles/PMC2682512>

2. Taherzadeh, G., Dehzangi, A., Golchin, M., Zhou, Y. and Campbell, M.P. (2019) SPRINT-Gly: predicting N- and O-linked glycosylation sites of human and mouse proteins by using sequence and predicted structural properties. *Bioinformatics (Oxford, England)*, **35**, 4140–4146.

<http://www.ncbi.nlm.nih.gov/pubmed/30903686>

<http://dx.doi.org/10.1093/bioinformatics/btz215>

3. Pitti, T., Chen, C.T., Lin, H.N., Choong, W.K., Hsu, W.L. and Sung, T.Y. (2019) N-GlyDE: a two-stage N-linked glycosylation site prediction incorporating gapped dipeptides and pattern-based encoding. *Scientific reports*, **9**, 15975.

<http://www.ncbi.nlm.nih.gov/pubmed/31685900>

<http://dx.doi.org/10.1038/s41598-019-52341-z>

<http://www.ncbi.nlm.nih.gov/pmc/articles/PMC6828726>

4. Chien, C.-H., Chang, C.-C., Lin, S.-H., Chen, C.-W., Chang, Z.-H. and Chu, Y.-W. (2020) N-GlycoGo: predicting protein N-glycosylation sites on imbalanced data sets by using heterogeneous and comprehensive strategy. *IEEE Access*, **8**, 165944–165950.

5. Pugalenthi, G., Nithya, V., Chou, K.C. and Archunan, G. (2020) Nglyc: A Random Forest Method for Prediction of N-Glycosylation Sites in Eukaryotic Protein Sequence. *Protein and peptide letters*, **27**, 178–186.

<http://www.ncbi.nlm.nih.gov/pubmed/31577193>

<http://dx.doi.org/10.2174/0929866526666191002111404>

6. Pakhrin, S.C., Aoki-Kinoshita, K.F., Caragea, D. and Kc, D.B. (2021) DeepNGlyPred: A Deep Neural Network-Based Approach for Human N-Linked Glycosylation Site Prediction. *Molecules (Basel, Switzerland)*, **26**, epublish.

<http://www.ncbi.nlm.nih.gov/pubmed/34885895>

<http://dx.doi.org/10.3390/molecules26237314>

<http://www.ncbi.nlm.nih.gov/pmc/articles/PMC8658957>

7. Alkuhlani, A., Gad, W., Roushdy, M. and Salem, A.-B.M. (2022) Pustackngly: positive-unlabeled and stacking learning for n-linked glycosylation site prediction. *IEEE Access*, **10**, 12702–12713.

8. Pakhrin, S.C., Pokharel, S., Aoki-Kinoshita, K.F., Beck, M.R., Dam, T.K., Caragea, D. and Kc, D.B. (2023) LMNglyPred: prediction of human N-linked glycosylation sites using embeddings from a pre-trained protein language model. *Glycobiology*, **33**, 411–422.

<http://www.ncbi.nlm.nih.gov/pubmed/37067908>

<http://dx.doi.org/10.1093/glycob/cwad033>

9. Hou, X., Wang, Y., Bu, D., Wang, Y. and Sun, S. (2023) EMNGly: predicting N-linked glycosylation sites using the language models for feature extraction. *Bioinformatics (Oxford, England)*, **39**, ppublish.

<http://www.ncbi.nlm.nih.gov/pubmed/37930896>

<http://dx.doi.org/10.1093/bioinformatics/btad650>

<http://www.ncbi.nlm.nih.gov/pmc/articles/PMC10627407>

10. Hu, F., Gao, J., Zheng, J., Kwoh, C. and Jia, C. (2024) N-GlycoPred: A hybrid deep learning model for accurate identification of N-glycosylation sites. *Methods (San Diego, Calif.)*, **227**, 48–57.

<http://www.ncbi.nlm.nih.gov/pubmed/38734394>

<http://dx.doi.org/10.1016/j.ymeth.2024.05.002>

11. Wang, H., Zhao, L., Yu, Z., Zeng, X. and Shi, S. (2025) CoNglyPred: Accurate Prediction of N-Linked Glycosylation Sites Using ESM-2 and Structural Features With Graph Network and Co-Attention. *Proteomics*, **25**, e202400210.

<http://www.ncbi.nlm.nih.gov/pubmed/39361250>

<http://dx.doi.org/10.1002/pmic.202400210>

12. Nafi, M.M.I. and Rahman, M.S. (2025) StackGlyEmbed: prediction of N-linked glycosylation sites using protein language models. *Bioinformatics advances*, **5**, vbaf146.

<http://www.ncbi.nlm.nih.gov/pubmed/40630498>

<http://dx.doi.org/10.1093/bioadv/vbaf146>

<http://www.ncbi.nlm.nih.gov/pmc/articles/PMC12237515>

13. Feng, Z., Zhang, X., Wang, H., Hong, X., Zhan, J. and Zhou, Y. (2025) ESM-LoRA-Gly: Improved prediction of N-and O-linked glycosylation sites by tuning protein language models with low-rank adaptation (LoRA). *bioRxiv*, 2025.2008. 2012.669850.

14. Touw, W.G., Baakman, C., Black, J., te Beek, T.A., Krieger, E., Joosten, R.P. and Vriend, G. (2015) A series of PDB-related databanks for everyday needs. *Nucleic acids research*, **43**, D364–368.

<http://www.ncbi.nlm.nih.gov/pubmed/25352545>

<http://dx.doi.org/10.1093/nar/gku1028>

<http://www.ncbi.nlm.nih.gov/pmc/articles/PMC4383885>

15. Elnaggar, A., Heinzinger, M., Dallago, C., Rehawi, G., Wang, Y., Jones, L., Gibbs, T., Feher, T., Angerer, C., Steinegger, M. *et al.* (2022) ProtTrans: Toward Understanding the Language of Life Through Self-Supervised Learning. *IEEE transactions on pattern analysis and machine intelligence*, **44**, 7112–7127.

<http://www.ncbi.nlm.nih.gov/pubmed/34232869>

<http://dx.doi.org/10.1109/tpami.2021.3095381>
